# Is intramedullary nailing more effective than non-operative treatment in adults with displaced middle-third clavicle fractures?

**DOI:** 10.1007/s10195-014-0299-6

**Published:** 2014-05-31

**Authors:** Christopher Edward Hill

**Affiliations:** 1 46, Hermitage Road, Kenilworth, Warwickshire CV8 2DW UK; 2 University Hospital of Coventry and Warwickshire, Clifford Bridge Road, Coventry, CV22 5PX UK

**Keywords:** Fracture, Clavicle, Nailing, Pinning, Intramedullary, Trauma

## Abstract

**Background:**

Clavicle fractures are common, accounting for 5–12 % of all fractures. Traditionally, displaced middle-third clavicle fractures have been managed non-operatively but the associated displacement often leads to mal-union with shortening, cosmetic deformity and occasionally non-union, with clinicians looking towards alternative operative methods such as intramedullary nailing (IMN). However, such methods have their own complications. In order to ascertain the effectiveness of IMN in the management of middle-third clavicle fractures compared with non-operative treatment, analysis of recent evidence is required and this review aims to achieve that, focusing on relevant, contemporary randomised-control trials.

**Materials and methods:**

Essential search-terms identified from the research question were used to formulate a search strategy. A systematic search of multiple databases was then performed from 1966 until present and appropriate papers for appraisal identified.

**Results:**

Thirteen papers were identified, with 10 excluded using appropriate eligibility criteria. The remaining papers were then critically appraised. With regards shoulder function, all papers demonstrated an association between IMN and a significantly (*P* < 0.05) superior shoulder function score, but no consensus with regards to complication rates. However, all have identified limitations; therefore, their overall findings must be considered conservatively.

**Conclusions:**

Further, high-quality research, ideally in the form of well-designed, multi-centre RCTs is required to allow acceptable implementation of IMN of middle-third clavicle fractures into widespread practice. However, early results demonstrate that in young patients with displaced middle-third clavicle fractures, who are motivated to return to work, IMN provides superior functional results and should be considered. However, the importance of considering each patient individually as to their suitability for each management option, before coming to an informed decision with the patient rather than having a blanket approach to MTCF is essential.

**Level of Evidence:**

Level 1.

## Introduction

This review aims to use clinically based, contemporary literature to ascertain whether intramedullary nailing (IMN) is a more beneficial management technique than non-operative management in adult middle-third clavicle fractures (MTCF) with regards to shoulder function.

Clavicle fractures account for 5–12 % of all fractures, with an estimated incidence of 29–64/100,000 adult population/year [1–4]. Eighty per cent occur in the middle-third zone of the clavicle, identified as Grade 1 using the anatomical Allman classification [5] (Table 1).

**Table 1:** The Allman classification of clavicle fractures

Grade 1:	Fractures of the middle-third of the clavicle
Grade 2:	Fractures of the clavicle distal to the coracoclavicular ligament (lateral)
Grade 3:	Fractures of the proximal end of the clavicle (medial)

Most MTCF are displaced by the deforming pull of associated muscle attachments [6–8]. Traditionally, MTCF have been managed non-operatively [9–11], however, the associated displacement often leads to mal-union with shortening, cosmetic deformity and occasionally non-union [1, 12, 13]. In connecting the upper limb with the thoracic cage, the clavicle is salient to shoulder mechanics and stabilising the shoulder girdle, hence fracture patients with loss of length and curvature have been reported to suffer with residual deficits in shoulder strength and endurance [1, 3, 12, 13].

These potential drawbacks demonstrate why MTCF management has become increasingly controversial, with clinicians looking towards alternative operative methods [1, 14]. Two main operative methods exist: internal plate fixation and IMN [15]. Internal plate fixation has been shown to have a number of complications, (Table 2), leading many to explore IMN [16]:

**Table 2:** Internal plate fixation complications [16, 54]

Internal plate fixation complications	
Deep infection	Implant failure
Loosening	Wide surgical exposure
Large scar/poor cosmesis	Neurovascular problem
Prominence	Non-/mal-union
Re-fracture	Need for metalwork removal

Clavicle IMN was initially described over 50 years ago [17, 18]. Biomechanically, the technique provides optimal fracture stabilisation and aims to preserve clavicular length, avoiding mal-union and maintaining good cosmetic and functional results, allowing faster return to daily activities and employment [19]. However, concerns regarding complication rates have raised doubts as to the best treatment method [17, 20]. Cochrane reviews have separately studied non-operative and operative treatment methods but not compared the two; therefore, given the fracture’s common incidence and management choice controversy, this review is fully justified [15, 21].

Evidence-based medicine is defined as “the conscientious, explicit and judicious use of current best evidence in making decisions about the care of individual patients” [22]. In order to ascertain the effectiveness of IMN in the management of adult MTCF compared with non-operative treatment, analysis of recent evidence is required and this paper aims to achieve that.

The Population, Intervention, Comparator and Outcome (PICO) process [23], was used to formulate the research question: in adults with middle-third clavicle fractures, is intramedullary nailing a more beneficial management technique than non-operative treatment with regards to shoulder function? The secondary outcome of complications was also investigated.

## Materials and methods

Having identified an appropriate, focussed research question, a search strategy was formulated with key concepts and keywords identified using the PICO process [23], identifying essential search-terms [24] which were exploded ensuring inclusion of relevant synonyms, alternative spellings and related terms. Individual search-terms were then combined using a Boolean technique [25, 26] to refine further.

Initial search keywords were broad, exploded terms to ensure full use of MeSH (Medical Subject Headings) terms for maximum sensitivity [26–28]. More specific terms and limitations were subsequently introduced and combined to refine the search [29]. Table 3 displays the full search strategy used.

**Table 3:** Literature search strategy example—medline (OVID)^®^ 1948 to date

**#**	Searches	Results	Search type
1	Nonoperative.mp.	6,003	Advanced
2	Non-operative.mp.	2,368	Advanced
3	Conservative.mp.	64,268	Advanced
4	1 or 2 or 3	71,706	Advanced
5	Exp Bone Nails/or exp Fracture Fixation, Intramedullary/or intramedullary.mp.	18,654	Advanced
6	4 and 5	506	Advanced
7	Clavicle.mp. or exp Clavicle/or clavicular.mp. or exp Clavicular/	6,448	Advanced
8	6 and 7	47	Advanced
9	Limit 8 to English language	29	Advanced
10	Limit 9 to humans	28	Advanced
11	Limit 10 to last 10 years	21	Advanced
9	Limit 11 to comparative study	6	Advanced

The search strategies for the remaining databases can be found in Appendix. Search performed 20 September, 2012

The eligibility criteria selected are displayed in Table 4.

**Table 4:** Inclusion and exclusion criteria

Criteria type	Description	Rationale for criteria
Inclusion criteria	Middle-third/midshaft clavicle fracture	Majority site for clavicle fractures
	Human subjects	Applicability to patients
	IMN as only defined intervention	As per research question
	Comparative study	To ensure papers selected for analysis were of a suitably high level in the evidence hierarchy [32] and allow comparison of intervention and comparator methods
	In English language	Deemed financially and temporarily inappropriate for this review for translation services of non-English papers despite potentially excluding relevant papers
	Published within last 10 years	To gain the most contemporary evidence available given scope of review
Exclusion criteria	Medial/lateral end of clavicle fracture	Rarer, uncommon type of fracture requiring different treatment considerations
	Non-human subjects/animal studies	Applicability to patients
	Alternative intervention, e.g. plating	As per research question
	Non-comparative studies	To ensure papers selected for analysis were of a suitably high level in the evidence hierarchy [32] and allow comparison of intervention and comparator methods
	Languages other than English	Deemed financially and temporarily inappropriate for this review for translation services of non-English papers despite potentially excluding relevant papers
	Published prior to last 10 years	To gain the most contemporary evidence available given scope of review

Multiple databases were used to ensure a thorough search was performed as no single database can cover all the resources within a given field [27]. These were: Medline, EMBASE and Web of Knowledge. The reference lists of all RCTs identified were hand-searched to identify further relevant articles.

Following the search, three papers were selected for critical appraisal, which is defined as the “process of systematically examining research evidence to assess its validity, results and relevance, before using it to inform a decision” [31:1]. To ensure a systematic, logical and standardised approach to the appraisal, the CASP reviewer check-list was used [30].

## Results

The results of the database searches are displayed in Table 3 and Appendix.

The aforementioned search criteria identified 13 papers, with 10 excluded as detailed in Table 5.

**Table 5:** Excluded studies following database searches

Study	Reason for exclusion
Houwert et al. [1]	Alternative intervention of plate fixation
Assobhi [56]	Alternative intervention of plate fixation
Duan et al. [57]	Alternative intervention of plate fixation
McKee [58]	Alternative intervention of plate fixation
Wijdicks et al. [59]	Alternative intervention of plate fixation
Ferran et al. [60]	Alternative intervention of plate fixation
Proubasta et al. [61]	Alternative intervention of plate fixation, cadaver study
Simon [62]	Not comparative study
O’Brian and Savoie [63]	Not comparative study
Thompson [64]	Not comparative study

This left 3 papers for critical appraisal, presented in Table 6, hereon referred to as papers 1–3.

**Table 6:** Final papers identified for critical appraisal

Paper	Title	References
Paper 1	Elastic stable intramedullary nailing is best for mid-shaft clavicular fractures without comminution: results in 60 patients	Smekal et al. [19]
Paper 2	Acute operative stabilization versus non-operative management of clavicle fractures	Judd et al. [65]
Paper 3	Elastic stable intramedullary nailing versus non-operative treatment of displaced clavicular fractures—a randomized, controlled, clinical trial	Smekal et al. [66]

### Critical Appraisal

#### Paper 1

This single-centre, prospective, controlled trial compared elastic IMN with non-operative treatment of displaced MTCF in adults aged 16–85. The paper lacked a clearly-focussed PICO-adhering research question [23]. The study population was defined using detailed eligibility criteria, and the comparative treatment was well described, both of which demonstrate good study design. The intervention was lacking in detail and although a standardised surgical method was well described, no detail was provided regarding the operating surgeons, predisposing it to inter-surgeon variability and proficiency bias [31]. However, the thorough description of the surgical method allows reproducibility of the study, making it generalisable. The study controversially selects two primary outcomes: time-to-union, for which the assessment process was explained in detail; and clavicular shortening, for which the assessment method was lacking. Secondary outcomes to be assessed are stated, but no description of data collection was provided, weakening study strength.

The study compared the efficacy of an interventional treatment with a comparison treatment and therefore an RCT is the preferred study design [32]. Despite being a prospective, controlled study using a well-recognised randomisation technique, it is stated as not being a RCT, with little justification. This is an unclear statement, especially given the low level of present evidence, meaning a gold-standard RCT would be highly appropriate for this comparative-clinical question [32].

The allocation-concealment process was briefly described as a single-block random assignment. This is a recognised, standardised method of true randomisation, which is a positive. However, no further information was provided regarding who performed the randomisation, use of blinding, sequence generation or treatment allocation. There was no mention of computer-assisted randomisation, and no audit trail to ensure reliability of the process. This lack of detail impacts negatively on the study, especially given that the authors later state it was not a RCT, raising suspicion regarding the validity of the randomisation process. Using a variable-block method is less predictable and would have strengthened the allocation process [33]. A true randomisation process aims to prevent baseline confounding factors between study groups, ensuring they are well balanced and strengthening the study [34]. Despite the process ambiguity, there were no significant differences (*P* > 0.05) between the group demographics, increasing trial robustness.

A major flaw was the lack of blinding. Given the study’s nature, participant and radiographic-assessor blinding were not possible. However, blinding could have been employed for data collection at clinical assessment, shoulder function score (SFS) recording and at data and statistical analyses. This would have reduced the impact of observer or detection biases [35].

The description of data collection methods were variable; however, a thorough description of the assessment technique for the primary outcome time-to-union is provided. This is a difficult end-point to assess, but a clear definition is denoted, with a standardised, reproducible technique described. The study uses 4-weekly radiographs, and although pragmatic and reproducible (enhancing external validity), this method only allows calculation of the time-to-union to the nearest 4-week interval, bringing detrimental imprecision to the study.

Other outcomes are measured more reliably, using contralateral comparison on standardised radiographs for shortening and computer tomography (CT) measurements for non-/mal-union. This is commendable as CT is the gold-standard assessment for union discrepancies, and the shortening measurement method is a standardised technique shown to have high agreement with CT measurements and high repeatability [8, 36]. This makes the study reproducible, improving its external validity. Similarly, standardised, well-recognised SFSs are utilised [37, 38]. However, description of the data collection method is brief; a negative point. The SFS results were collected via patient questionnaires at 2 years, leading to non-responder and recall bias as well as placing heavy reliance on self-reporting, which often results in a high loss to follow-up [34, 39, 40]. However, no mention of this was detailed, with participants apparently accounted for throughout which, if true, is commendable in reducing the attrition bias effect [34]. However, this is difficult to achieve, thus failure to mention it leads to suspicion.

Details of patients lost to follow-up, excluded from or declining to participate in the trial are not provided. Inclusion of a CONSORT-type flow diagram [41] defining enrolment, allocation and follow-up numbers would resolve this and significantly strengthen the study. The authors disclose cross-over between treatment groups resulting in contamination bias [42]. However, only per-protocol analysis is conducted with no intention-to-treat analysis, which would have reduced the impact on the randomisation process and avoided selection bias [35]. This significantly weakens the study as intention-to-treat analysis would have provided the most conservative estimate of relative effect size, thus demonstrating the most reliable significant difference if found, despite the cross-over. Comparison of both analyses should have been performed as per-protocol analysis alone may distort the evidence [43].

Another study weakness was discrepancies in the group’s follow-up, with the non-operative group unable to begin mobilising until 3 weeks post-injury compared with immediate post-operative mobilisation in the IMN group. Although difficult to assess, this may have introduced performance bias [34], affecting shoulder stiffness or healing rates. However, the outcome methods for the groups were the same.

The lack of a sample-size calculation is a significant weakness, as achieving a statistically calculated sample size increases study strength due to increased power and probability that a significant difference will be discovered [44]. Instead, the overall sample size is small with the study underpowered, more prone to Type II error, and hence less likely to find a significant difference [45]. Conducting the trial over multiple centres would have improved this, as well as increasing external validity.

The study concludes that in patients with MTCF, when compared with non-operative management, IMN leads to significantly (*P* < 0.05) better shoulder function at 2 years follow-up, as well as faster time-to-union, lower non- and delayed union rates and less clavicular shortening. However, it found no significant difference in the total number of complications between groups.

#### Paper 2

This single-centre RCT assessed IMN with non-operative treatment in adult patients aged 17–40 with isolated MTCF. The study aimed to compare the efficacy of an intervention with a comparison treatment, hence a RCT is the preferred study design [32], with the topic remaining relevant.

The paper fails to identify a clear research question at the outset, making trial specifics difficult to ascertain. When assessing PICO methodology [23] the population is clearly defined using detailed eligibility criteria, demonstrating strong study design. However, criterion justification is lacking, e.g. ages 17–40. This is especially relevant given that MTCFs have a bimodal age distribution, occurring in the young adult and ages 55–75 [46, 47]: with the latter therefore excluded. This introduces sample bias and substantially reduces the study’s generalisability and external validity as extrapolation to the older subgroup cannot be reliably performed. The authors’ affiliated institution is an Army Medical Centre. Therefore, the reason for this age criterion is likely due to a subgroup military population, a point not discussed but further evidenced by the demographic male majority and patient motivation to return to “duty”. If true, this should have been openly stated as this subgroup does not reflect the general population, further reducing external validity and generalisability.

The intervention and comparison techniques are well described, strengthening the study and enhancing reproducibility. Limited details regarding the operating surgeons are provided, however, which could mask inter-surgeon variability and proficiency bias [31]. There are discrepancies between the time-to-theatre, (0–2 weeks), thus the amount of bone-healing underway at the time of surgery is variable which could affect results. Importantly this leads to a degree of cross-over and contamination bias [42], as a third of participants in the operative group underwent up to 2 weeks non-operative management before surgery. This could be long enough for significant fracture callus formation [48, 49], potentially predisposing union discrepancies. However, no consideration is given to this merging of the intervention and comparison techniques, reducing the likelihood of significant differences being found. Thus, results must be interpreted conservatively.

The outcomes are not clearly stated. Only on reaching the discussion section is “the goal” of the study detailed, implying the outcomes are SFS and non-union rate. Standardised, validated SFSs are used which is a positive due to their reliability, availability and validity [50, 51], as well as ensuring the study’s reproducibility and generalisability. However, because they are patient-reported questionnaires they do carry the aforementioned negatives of non-responder and recall bias.

The randomisation process description is inadequate. There is no detail regarding how the initial randomisation was generated or who was conducting the randomisation and allocation processes. Hence this remains a questionable method of true randomisation with a lack of independent audit trail, leaving it open to potential tampering [33]. Analysing group demographic data for significant differences can assess whether the randomisation process has overcome confounding factors: something this paper did not perform, another negative point.

The same blinding issues are true here as in paper 1, weakening the study by exposing it to detection bias [35], with the aforementioned improvements to study design relevant.

The methods of data collection are relatively well described, with SFS questionnaires completed at initiation and at regular intervals up to 1 year post-injury, allowing progress monitoring. However, secondary outcome assessment methods for union and shortening were less reliable. Positives were standardised X-rays for each participant, reducing inter-participant variability regarding discrepancies in rotation or magnification on X-rays, and separate examiners performing radiographic measurements and averaging their individual findings for an overall result with increased accuracy. However, standard rulers and goniometers were used, both of which are open to instrument and assessor bias [52]. Also, the definition given for “healing” (union final outcome point) was ambiguous, defined as “callus across the fracture site”, with no criteria provided. This lack of precision will lead to assessor inter-variability, contributing to decreased accuracy, as well as making the overall study less reproducible, reducing its generalisability.

The study lacks a CONSORT-type flow diagram [41] and provides little information regarding the numbers of participants involved. It is stated that 57 enrolled, but no details are given concerning the overall number approached, participants changing treatment group from their random allocation, or any being lost to follow-up. If true, then both the latter strengthen the study considerably, but should not be assumed.

A positive point was the use of identical follow-up for both groups. This reduces treatment method confounding factors, and allows assessment of their pure effect more accurately. However, few details regarding post-treatment rehabilitation are provided, decreasing reproducibility and external validity. If rehabilitation involved intense, regular physiotherapy sessions, this may not be generalisable to most healthcare systems where multiple factors make this unfeasible.

There is no power calculation and the sample size is small, the negatives of which have been discussed previously. In the text, limited result information is provided and not easily extrapolated, e.g. SFS showed a significant difference (*P* < 0.04) at 3 weeks, but analysis to 1-year follow-up was not provided. Thus, the study temporally limits itself, and does not denote whether this difference is maintained long-term: information that is essential when considering the techniques for use in the general population, which is a major weakness.

The study concludes that in young adults with MTCF, when compared with non-operative management, IMN gives superior SFS at 3 weeks, no significant difference in union rates, but a higher overall complication rate.

#### Paper 3

This single-centre RCT compared elastic IMN with non-operative treatment of displaced MTCF in patients aged 18–65. As previously discussed, a RCT remains the preferred study design [32]. Although the paper confirmed its aim, there was no fully PICO-adherent research question [23]. Thorough eligibility criteria are provided, accurately defining the study population, though criteria justification was lacking. Age was again limited, with the aforementioned disadvantages remaining relevant. Treatment methods were described in detail, following standardised techniques allowing reproducibility, enhancing external validity. However, there was again a lack of detail regarding the surgeons operating. Outcome measurements were clearly identified and detailed, strengthening the study, enhancing readability and reproducibility. However, none were designated a priori, a limitation identified by the authors, suggesting they deduced the outcomes retrospectively.

The randomisation process was well described, using an accepted standardised balanced 4-block randomisation method. The paper excels where the others failed in providing specifics regarding the allocation process, detailing how the randomisation sequence was generated, allocated and by whom, enhancing study strength. However, the staff generating and allocating the randomisation sequence were the surgeons involved in the study, introducing bias and demonstrating a lack of blinding, a theme continued throughout the paper. This lack of an independent, external party and a defined audit trail reduces the process validity, leaving it exposed to tampering, resulting in a less robust trial design. The fixed-block randomisation method was somewhat predictable, especially as the block-size was known to the surgeons, and a variable-block randomisation would have been superior [33]. The assigned treatment options of patients lost to follow-up were re-used in an attempt to maintain the original randomisation, but via a questionable method. Generating larger numbers of randomisation options initially with the allowance for drop-outs would have been more valid [33]. Despite these limitations, no significant differences were found between the group demographics, a positive point in removing confounding factors and allowing a fairer comparison [34].

The SFS outcomes used were the DASH and Constant scores which are validated, well-recognised, responsive, readily-available, reproducible scores [37, 38]. The DASH questionnaires were assessed weekly for the first 6 months, allowing close observation of participant progression. However, due to expense and practicality, patients were seen monthly thereafter where four questionnaires were collected. This pragmatic approach introduces the risk that patients may complete all forms together retrospectively, an identified compliance limitation. The Constant score is used for the SFS at 6 and 24 months, though no justification is given for its replacement of the DASH score at this stage, which is especially relevant as the DASH scores demonstrated significant differences up to, but not after 18 weeks, whereas the Constant score showed significant differences at 6 and 24 months. Use of both scores throughout the follow-up would have increased the reliability and validity of the result, but would be less pragmatic, and may lead to increased loss to follow-up along with the aforementioned bias issues associated with questionnaire use [34, 39, 40].

The radiological evaluation methods were well described, strengthening the study. Regular, standardised X-rays were used to reduce inter-patient variability and increase the chance of pinpointing the moment of union. However, no assessor details were provided and definitions of end-points were vague and non-reproducible, reducing external validity. Use of CT was employed if there was no obvious union after 24 weeks, which is a previously mentioned positive. However, surgery was then offered to those with a confirmed non-union. Given that follow-up lasted 2 years, this may have led to contamination bias and cross-over. Ten patients developed non-union, but no details regarding further surgery performed are provided. If significant cross-over did occur, this will bias results and appropriate intention-to-treat and per-protocol analyses for outcomes after that period should be performed and compared as discussed previously.

This paper provides good detail regarding patient numbers, including patients excluded, those declining to participate and those lost to follow-up. However, no sample-size calculation was performed and the study is underpowered, increasing the likelihood of false-negative results as previously discussed [44, 45].

The paper concludes that in adults with MTCF, when compared with non-operative management, IMN demonstrated significantly (*P* < 0.05) better SFS, less shortening, fewer complications and shorter time-to-union.

## Discussion

MTCFs are common and traditionally treated non-operatively [9–11]. Although this can be successful, recent studies have shown it can lead to serious cosmetic and functional complications [1, 3, 12, 13]. IMN presents a biomechanically sound alternative and potentially avoids many of the aforementioned complications, boasting superior functional results earlier and a faster return to normal activities [19]. However, some studies have shown higher complication rates [17, 20]. This review appraised the contemporary evidence, with results comparing IMN with non-operative management in adult MTCF to ascertain which is more beneficial for shoulder function (primary objective) and regarding complication rates (secondary objective); summarised in Table 7:

**Table 7:** Summary table of the reviewed papers based on PICO methodology [23]

Study	Population	Intervention	Comparison	Stated outcome/findings
Paper 1: Smekal et al. [19]	112 participants (age 18–65)	Elastic stable intramedullary nailing (*n* = 60)	Non-operative management (sling) (*n* = 52)	IMN demonstrated significantly (*P* < 0.05) better shoulder function at 2 years follow-up, faster time-to-union, lower non- and delayed union rates and less clavicular shortening
				No significant difference in the total number of complications between groups
Paper 2: Judd et al. [65]	57 participants (aged 17–40)	Intramedullary nailing (Hagi pin) (*n* = 29)	Non-operative management (sling) (*n* = 28)	IMN demonstrated significantly (*P* < 0.04) superior SFS at 3 weeks
				Higher overall complication rate (though significance testing not performed)
				No significant difference in union rates
Paper 3: Smekal et al. [66]	60 participants (aged 18–65)	Elastic stable intramedullary nailing (*n* = 30)	Non-operative management (sling) (*n* = 30)	IMN demonstrated significantly (*P* < 0.05) better SFS, less shortening, fewer complications and shorter time-to-union

With regards to shoulder function, all three papers demonstrated an association between IMN and a significantly (*P* < 0.05) superior SFS. However, all are flawed. Paper 1 only provides functional scores at 2 years, and cannot provide information regarding early post-operative weeks, thus it cannot assess early return to daily activities. Conversely, paper 2 calculated significance scores up to 3 weeks. Paper 3 showed an initial significant difference, but this declined until no longer significant at 18 weeks onwards, after which the SFS used was switched, then showing a significant long-term difference. All 3 papers had multiple limitations, therefore overall findings must be considered conservatively and further research in the form of well-designed RCTs is required. On balance, however, results appear to show IMN as producing a better functional outcome than non-operative management.

When considering the secondary objective of complications, the results are less conclusive, (findings shown in Table 7). Although paper 1 has a higher power, it displayed numerous flaws, especially compared with paper 3, which demonstrated stronger study design. The underpowered nature of papers 2 and 3 mean they cannot be considered singularly conclusive, highlighting the fact that further research is required, with complications individually identified in order to truly assess them in the interests of patient safety [53].

When considering the implications for future clinical practice, the overall external validity is salient. Clavicle fractures have a bimodal age distribution [46, 47], but all papers limited the trial population age, excluding most or all of the older group, reducing generalisability, as findings cannot be universally extrapolated. Nevertheless, the remaining aspects of the trials are reproducible, with detailed techniques and assessment methods provided and widely available implants used, adding to study external validity, making it feasible to implement into practice. However, the effect of the aforementioned limitations must be considered before proceeding.

A salient factor to practice is cost, which none of the papers discussed. Initial surgical costs are higher than non-operative treatment, hence operative treatment of all MTCFs would substantially increase management costs, although if it resulted in a faster return to work then the financial benefits gained could justify this. The cost-effectiveness of operating also depends on the longevity of the functional advantage gained [54], therefore highlighting the need for further research evaluating the cost-benefit analysis of treatment methods, especially given the current need for greater accountability in healthcare spending.

Certain fracture-patterns of MTCFs may show an increased benefit compared to others when comparing the two treatment methods, which papers 1 and 3 alluded to by sub-dividing the MTCFs into simple, wedge and comminuted/complex fractures. This showed IMN had the most significant improvement in functional scores in comminuted fractures, despite the higher incidence of shortening, due to stabilisation allowing a faster rehabilitation, and hence a better long-term outcome. This highlights another area for further research to identify how fracture sub-groups progress with IMN, hence identifying those that could be still managed non-operatively, reducing costs and thus making enrolment into practice more achievable.

In papers 1–3 a randomisation method has been used for treatment allocation. Although this is appropriate in a trial setting, for clinical practice a more considered approach must be implemented based on the best evidence available. Each patient must be assessed individually for their suitability to each management option before coming to a decision with the patient, rather than a blanket approach being adopted. As well as the abovementioned factors of age and fracture configuration, patient factors such as co-morbidities, expectations, occupation and activity level will have an influence on the treatment type selected and must be considered when determining the treatment option.

Evidence-based medicine involves appraisal, evaluation and judicious use of the current best evidence to make appropriate decisions about the care of individual patients [23]. When considering the initial research question, all the papers demonstrated a significant (*P* < 0.05) advantage of IMN over non-operative management in displaced MTCF with regards to shoulder function, but no consensus with regards to complication rates. All conclusions drawn need to be viewed conservatively due to the aforementioned limitations, in particular the age restrictions. Further, high-quality research addressing the aforementioned issues, ideally in the form of well-designed, multi-centre RCTs is required to allow acceptable implementation of IMN of MTCF into widespread practice. However, early results demonstrate that in young patients with displaced MTCF, who are motivated to return to work, IMN provides superior functional results and should be considered. However, the importance of considering each patient individually as to their suitability for each management option, before coming to an informed decision with the patient rather than having a blanket approach to MTCF is essential.

## Appendix: Literature search strategies

All searches performed 20 September, 2012.

### EMBASE

**Table:**

1. Conservative treatment/or nonoperative.mp.	48,623
2. Exp conservative treatment/or non-operative.mp.	355,898
3. 1 or 2	361,883
4. Exp intramedullary nailing/or exp intramedullary nail/or intramedullary.mp.	20,922
5. 3 and 4	1,122
6. Exp clavicle fracture/or exp clavicle/or clavicle.mp.	8,871
7. 5 and 6	62
8. Limit 7 to English language	46
9. Limit 8 to human	44
10. Limit 9 to last 10 years	41
11. Limit 10 to clinical trial	7

### Web of knowledge

**Table:**

1. Intramedullary/AND clavicle/AND fracture	168
2. Limit 1 to English language	105
3. Limit to clinical trial	7
